# Application of checklist-based nursing care process in patients undergoing intervention for coronary chronic total occlusions: a quasi-randomized study

**DOI:** 10.1186/s12872-023-03627-8

**Published:** 2023-11-30

**Authors:** Xia Ge, Haiyang Wu, Zhe Zang, Jiayi Xie

**Affiliations:** Cardiovascular ward, Shengjing Hospital of China Medical University, Shenyang, 110004 Liaoning China

**Keywords:** Coronary chronic total occlusions, Checklist, Percutaneous coronary intervention, Nursing

## Abstract

**Background:**

Coronary chronic total occlusion (CTO) interventions are more complex than general percutaneous coronary intervention (PCI) procedures. However, only a few nursing methods are specifically applied to patients undergoing CTO interventions. And the conventional nursing effect is not ideal, urgent need to explore more effective nursing methods. The checklist is a simple and effective tool for error management and performance improvement that has been widely used in many fields. But there have been no reports of the checklist being used to improve care for CTO patients.

**Objective:**

This study aimed to investigate the effectiveness of a checklist-based nursing care process in patients undergoing Coronary chronic total occlusion (CTO) interventions, including duration of care, patient anxiety, improved patient satisfaction, and occurrence of adverse events.

**Methods:**

A total of 120 CTO patients undergoing percutaneous coronary intervention (PCI) were selected at our hospital and divided into an intervention group (*n* = 60, adopted the checklist-based nursing care process for patient care) and a control group (*n* = 60, adopted nursing care according to the existing workflow) according to different nursing interventions. After surgery, the nurse in charge of the patient completed the nursing according to the “List of postoperative care for CTO patients” filled in by the patient within 24 h after surgery, conducted a doctor satisfaction survey, recorded adverse events, and completed the postoperative Self-Rating Anxiety Scale (SAS) score and patient satisfaction survey before the patient was discharged. Subsequently, the Qc team checks the completion of the patient’s checklist for safety and the completion of the questionnaire. Finally, the differences between the two groups in preoperative nursing time, incidence of adverse events caused by nurses’ omission or inadequate guidance, patient anxiety, and doctor and patient satisfaction were compared.

**Results:**

The intervention grouphad significantly shorter preoperative nursing care time and significantly lower the total number of adverse events than the control group (*P* < 0.05).The postoperative Self-Rating Anxiety Scale (SAS) score of the intervention group was significantly lower than that of the control group (*P* < 0.05).The satisfaction of doctors and patients in the intervention groupwas significantly higher than that in the control group (*P* < 0.05).

**Conclusion:**

The application of the checklist-based nursing care process in patients with CTO intervention can significantly reduce the preoperative nursing care time, reduce patient anxiety, improve patients’ and doctors’ satisfaction with nursing care, and effectively reduce the occurrence of adverse events caused by nurses’ omissions or inadequate instructions.

**Trial registration:**

The protocol of the trial was registered retrospectively of Chinese Clinical Trial Registry (registration number ChiCTR2200056804, reg date17/02/2022).

## Background

Coronary chronic total occlusion (CTO) is a common type of lesion in patients with coronary artery disease, and percutaneous coronary intervention (PCI) serves as one of the primary treatment methods for CTO patients [1–4]. Compared to other modalities such as drug therapy [4–6], PCI procedures offer significant advantages. Several studies have reported that CTO lesions account for 18–52% of patients undergoing coronary angiography [7–9]. However, patients with CTO face more severe vascular lesions compared to patients with common coronary artery disease, typically requiring longer surgeries and extending postoperative recovery time. Nurses must undergo more extensive preparation before the surgery. The postoperative nursing process and education for CTO patients also have obvious characteristics. This complicates and reinforces the care required during the PCI process [10]. Various nursing methods have been applied to PCI surgery, including Orem self-care mode [11], evidence-based care [12], full course care [13], and clinical nursing pathway [14]. However, most of these studies focus on the benefits for patients, There is still a lack of analysis and exploration on improving nursing work efficiency, reducing nursing defects and adverse events. Therefore, the nursing approaches typically used for PCI procedures may not be entirely suitable for patients with CTO [11–14].

Traditionally, PCI for non-occlusive disease was considered a low-risk procedure for patients with stable coronary artery disease. A national audit of over 100,000 PCIs conducted annually reported early Q-wave MI, emergency CABG, cerebrovascular accident, and mortality rates at 0.03%, 0.02%, 0.04%, and 0.14%, respectively36. However, significant advancements in coronary intervention equipment now allow successful revascularization of a broad range of CTO lesions using advanced techniques. Performing CTO interventions safely requires specialized training and education in centers committed to achieving a high level of success for these challenging procedures [10].

When performing interventions in hospitalized patients with CTO, various nursing-related challenges arise [11–14]. Furthermore, the absence of a unified and standardized management process indirectly impacts clinical work and reduces the satisfaction of both doctors and patients [11–14]. As routine nursing practices may not yield satisfactory results, it is essential to explore more effective nursing methodologies [11–14]. Checklists have been proven to be a simple and effective tool for error management and performance improvement in fields such as biology, education, and food service [15–17]. Widely recommended for use in the medical field, checklists have demonstrated significant results in the operating room [18], intensive care unit [19], obstetrics [20], dialysis [21], and medical teaching [22]. Despite this, checklists have not been reported to improve the care of CTO patients.

Therefore, this study aims to evaluate the application of checklist-based nursing care for patients undergoing CTO interventions by analyzing various indicators, including the duration of care, patient anxiety levels, improved patient satisfaction, and occurrence of adverse events.

## Methods

### Study design

**Fig. 1:**
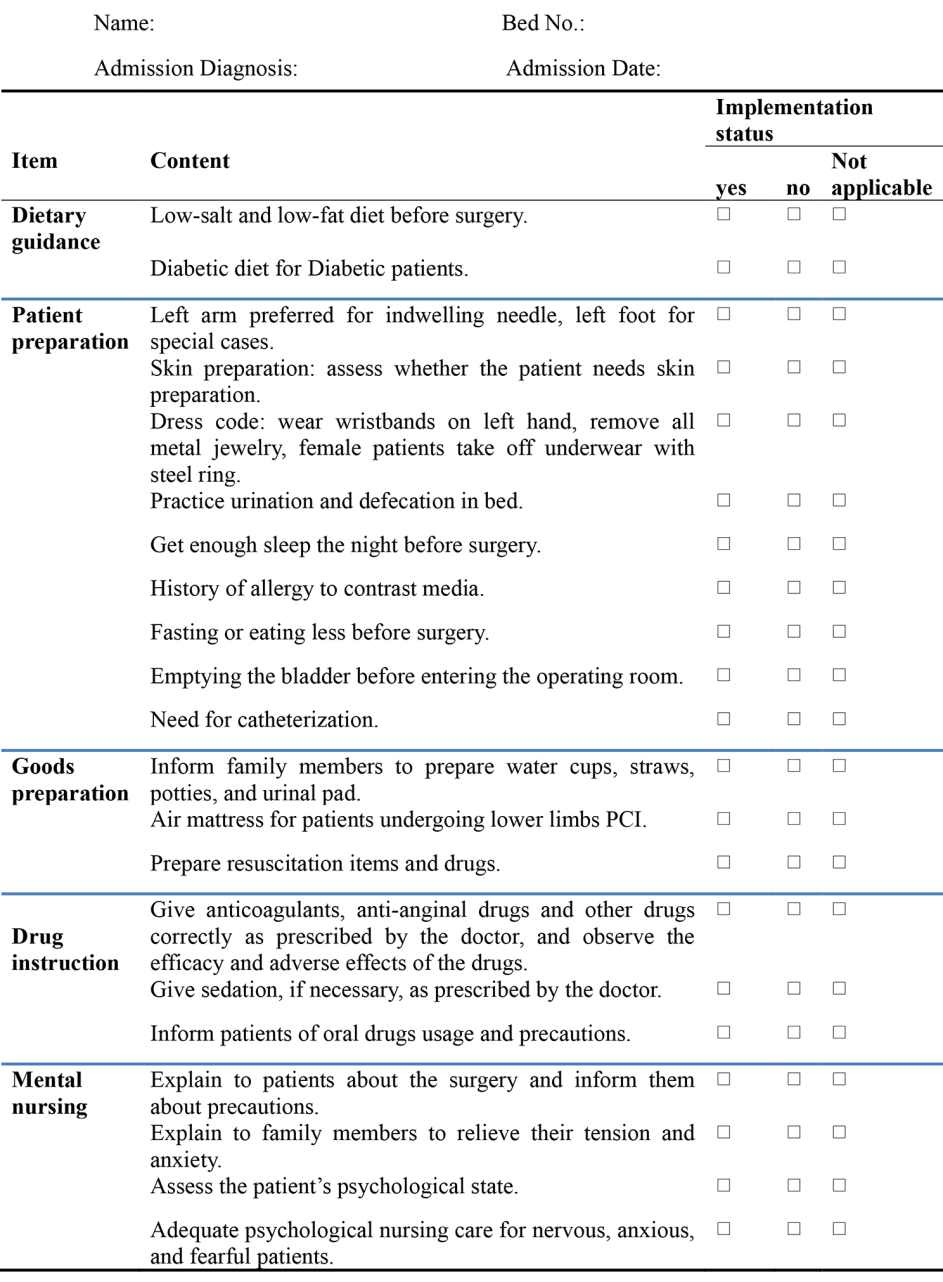
Preoperative-PCI nursing care checklist for CTO patients

Our study began with the formulation of the checklist.The design of the checklist was based on the latest version of the 2020 Chinese Interventional Guidelines, CTO Guidelines, and related literature, combined with the current situation of the department where the work was conducted, to develop a list of nursing tasks before and after CTO interventions in line with clinical reality. The contents of the checklist were subjected to expert correspondence by the Delphi method [40] (The premise of this method is that pooled intelligence can enhance individual judgement. In practice, the researcher choses the panel of experts, and develops a series of iterative questionnaires. Panelists reply anonymously to the iterative questionnaires, where every questionnaire sent represent a round. At every round, panelists receive feedback in the form of a statistical representation of the overall group’s response. The goal of the multiple iterations is to reduce the range of responses and gain consensus based on criteria chosen a priori by the researcher. The critical issues of conducting a Delphi study are the development of the questionnaire, the definition of consensus and the interpretation of non-consensus, criteria for selection of the panel and data analysis) [40], and the final contents of the list were determined (Fig. 1). Nursesresponsible for the overall care of patients will then assist 120 CTO patients recruited from December 2020 to July 2021 to complete the checklist. The main intervention target, the nurse, can improve the quality and efficiency of work by using the checklist, and finally achieve patient satisfaction and doctor satisfaction.

### Sample and setting

Patients who underwent CTO intervention in the Department of Cardiology, Shengjing Hospital, China Medical University, in Shenyang, China, from December 2020 to July 2021, were selected.The inclusion criteria were as follows: [1] the clinical symptoms and coronary angiographic results were in accordance with the international diagnostic criteria for CTO [2]; age ≥ 18 years and the disease was within the scope of indications for intervention [3]; physically able to receive cardiac treatment interventions [4]; no relevant contraindications [5]; normal cognition, hearing and intelligence, and basic communication and understanding ability; and [6] complete data collection, voluntary participation, and good compliance in this study.

The exclusion criteria were as follows: [1] patients with serious chronic diseases or major organ dysfunctions such as diseases of the liver and kidney [2]; patients with unopened vessels requiring secondary surgery or bypass [3]; patients who are not undergoing PCI for the first time [4]; patients who cannot communicate effectively [5]; patients with psychiatric disorders, psychiatric history, visual and hearing impairment, or cognitive impairment [6]; patients who develop serious mental or physical illnesses during hospitalization; and [7] patients who are breastfeeding or pregnant.

### Interventions

Intervention group: The checklist-based nursing care process was used to provide care and education to patients with CTO before and after PCI, and the specific contents and methods are as follows. A paper version of the checklist-based nursing process required for the study was also printed. After the patient with CTO is admitted to the hospital, the primary nurse will create a checklist for him/her, fill in the complete patient information, and manage the admitted patient according to the checklist item by item, that is, tick “√” at “yes” for completed tasks and “√” at “no” for those that are not completed. If the patient fails to complete this item during the implementation process, tick “√” at “not applicable,” and the checklist will be kept for statistical purposes after the patient is discharged from the hospital. Within 24 h of admission, the primary nurse will score the patient based on the Self-Rating Anxiety Scale (SAS) and provide nursing care to the patient following the “Preoperative Nursing Care Checklist for Patients with CTO.”

**Fig. 2:**
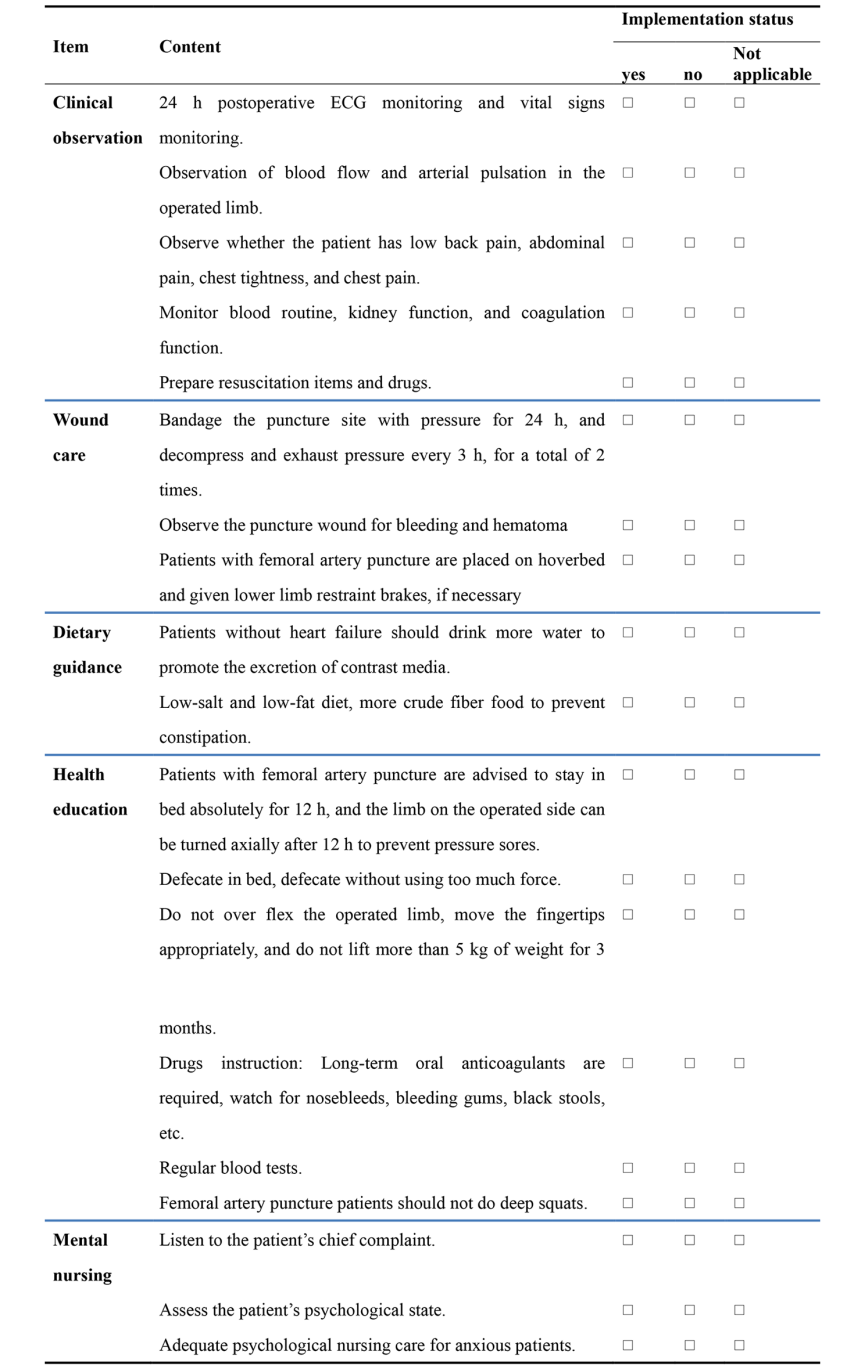
Postoperative-PCI nursing care checklist for CTO patients

Nurses need to be trained on intervention prior to intervention.All contents of the preoperative nursing care checklist for patients with CTO should be completed 1 day before surgery to 1 h before surgery (Fig. 1). The patients only fill in demographic details at the top of the checklist. The rest is done by the nurse.The members of the quality control team should check whether all contents of the checklist and the questionnaire are completed within 1 h before surgery and inform the primary nurse in time if there are any missing items and make up for them before surgery. The primary nurse should record the time needed to complete all the contents of preoperative nursing checklist.After surgery, the primary nurse provided care to the patient following the “CTO patient postoperative nursing care list,” completed the postoperative nursing care checklist for patients with CTO within 24 h after surgery (Fig. 2), conducted doctor satisfaction survey, recorded adverse events (see Fig. 2), completed the postoperative SAS scale scoring before patient discharge, and conducted the patient satisfaction survey. Members of the quality control team checked the completion of the checklist before the patients were discharged and checked the completion of various questionnaires. In case of any omission, the primary nurse shall make up in time.And also, not all differences between groups are reflective of the use of the checklist.

Control group: Nursing care process for patients with CTO was carried out according to existing working methods, clinical observation, and health education. That is, nurses performed preoperative preparation and provided postoperative nursing care according to medical advice and personal work habits after patients were admitted to the hospital, placed intravenous indwelling needles one day or on the same day before surgery, performed skin preparation in the operating area, and provided preoperative diet, medication, and psychological guidance. The time required to complete all preoperative nursing tasks was recorded. The patient’s clinical observation, wound care, postoperative education, diet instruction, medication, and psychological guidance were conducted regularly according to the doctor’s postoperative advice. The preoperative and postoperativeSASscoring was performed within 24 h of admission and before discharge, respectively. The doctor satisfaction survey was conducted after surgery. The patient satisfaction survey was performed before discharge, and the occurrence of adverse events was recorded.

Quality control: [1] A checklist for quality control team was also established. The head nurse served as the team leader, and the team members were the head nurse assistant and two senior primary nurses with more than 5 years of experience. The team members were trained with the contents of the checklist, overall process of the study, and when to conduct checks to ensure the implementation of the checklist. The quality team measuring the occurrence of adverse events was blinded to the study allocation groups for the patient outcomes they were assessing [2]. Training organization. The head nurse organizes trainings for all nurses in the department to study the content of the checklist, understand the overall process of scientific research, and ensure complete data collection [3]. Implementation of supervision and inspection. Members of the quality control team strictly check whether the study content is implemented on time before and after the surgery, and in any case of omission, the primary nurse is required to further complete the tasks to ensure appropriate implementation.

### Measurements

Preoperative nursing care time. This refers to the sum of time spent by the primary nurse on all matters related to preoperative preparation, which was timed by the primary nurse herself.The less total preoperative nursing care time, the less the work time.

Occurrence of adverse events due to nurse’s omission or inadequate instruction. This includes inadequate preoperative patient preparation (failure to leave a venous access or incorrect placement of the venous access, blockage of the venous access, failure to prepare the skin in the operative area, and omission of preparations), failure to clean the contaminated operative area during surgery (such as vomitus and feces), and failure to train the patient about defecation on bed after surgery resulting in postoperative urinary difficulties, puncture site injury, bleeding, hematoma, and skin problems in patients after braking of the femoral artery puncture.

Patients’ anxiety. The SAS scale was used to compare the preoperative and postoperative anxiety of the two groups. There were 20 items in total, and the 4-level scoring methodwas adopted, with 15 positive scores and 5 negative scores.<50 points indicate no anxiety, 50–59 points indicate mildanxiety, 60–69 points indicate moderate anxiety, and > 69points indicate severe anxiety [41]. The higher the score, theworse the mental state [41].

Doctor and patient satisfaction. The “Doctors’ Satisfaction with Nursing Care Questionnaire” and the “Patients’ Satisfaction with Nursing Care Questionnaire” were used for the assessment. The higher the score, the better the satisfaction.

### Validity and reliability of instruments

Cronbach’s α of SAS was 0.932.The content validity index of the nursing care checklist for CTO patients was 0.82, and internalconsensus reliability Cronbach’s α coefficient was 0.859. The content validity index of theDoctors’ Satisfaction with Nursing Care Questionnairewas 0.85, and internalconsensus reliability Cronbach’s α coefficient was 0.893. Thecontent validity index of Patients’ Satisfaction with Nursing Care Questionnairewas 0.83, and internal consensus reliability Cronbach’s α coefficient was 0.898. All the above scales have good reliability and validity.

### Randomization

Doctors use random sequence software to generate 1 to 120 random serial numbers. Random serial numbers and then in the order in which the patients were treated, from front to back. Odd-numbered patients were included in the intervention group, while even-numbered patients were included in the control group. In order to avoid cross-infection during the experiment, the intervention group and the control group were arranged in wards A and B respectively. (Note: Both groups of patients were blinded, meaning they were unaware of whether they were in the experimental group or the control group; Doctors who evaluate the quality of patient care use a blind method, that is, they are unaware whether the evaluated patients are in the experimental group or the control group; The nurses in the control group used a blind method, that is, they were unaware that the ward had become the control group and still used existing working methods to care for patients; The nurses in the experimental group used a non blind method, that is, they were aware of using checklists to care for patients and received unified training.)

### Sample size

Group sample sizes of 43 and 43 achieve 90% power to reject the null hypothesis of equal means when the population mean difference of preoperative nursing care time is 5 min and - with a standard deviation for both groups of 7 min and with a significance level (alpha) of 0.050. Assuming a 20% potential dropout rate, the final sample size was increased to 120 subjects, with 60 subjects in each intervention group.

### Statistical analysis

SPSS 26.0 software was used for statistical analysis. Measurement data were expressed as (‾x ± s), and the t test was used for comparison between groups. Count data were expressed as number of cases or percentage, and the χ2 test was used for comparison between groups. The difference was considered significant at *P* < 0.05.

## Result

### The basic information between the two groups

Among the 120 patients, 109 completed the study, and 11 patients did not complete the questionnaire due to early discharge from the hospital. The final intervention group included 56 patients with mean age of (65.05 ± 10.14) years, and the control group included 53 patients with mean age of (65.02 ± 10.73) years. No significant difference was found between the two groups in terms of age, gender, ethnicity, marital status, job and health insurance type, and days of hospitalization (all P > 0.05; Table 1).

### Preoperative nursing care time

The average preoperative nursing care time in the intervention group was 37.61 ± 5.15 min, which was much lower than the 50.98 ± 5.61 min in the control group. In the intervention group, 71.4% and 28.6% of the patients had preoperative nursing time < 40 and 40–50 min, respectively, and all patients had preoperative nursing time < 50 min, compared with 3.8% and 37.7%, respectively, in the control group. In addition, 58.5% of patients had a preoperative care time > 50 min. The preoperative care time in the intervention group was significantly reduced compared with that in the control group, and the difference was significant (*P* < 0.05; Table 2).

### Occurrence of adverse events

Preoperatively, the intervention group had indwelling venous access in the wrong limb and one venous access blockage, while the control group had two non-indwelling venous accesses, two indwelling venous accesses in the wrong limbs, and three blocked venous accesses. Intraoperatively, vomiting and defecation did not occur in theintervention group, while one case of vomiting and two defecations were recorded in the control group. During the postoperative period, one case of bleeding at the puncture site without dysuria and skin-related problems occurred in the intervention group, while in the control group, one case of bleeding at the puncture site, two hematomas at the puncture site, two cases of dysuria, and one case of degree I pressure sore caused by braking after femoral artery puncture occurred. When each adverse event was compared separately,there was no significant difference (P > 0.05) except the “Omission goods”. However, the total number of adverse events, the occurrence of nursing-related adverse events in the intervention groupwas significantly lower than that in the control group (*P* < 0.05; Table 3).

### Patient anxiety

Most patients in both groups had different levels of anxiety before surgery, and the difference in SAS scores was not significant (P > 0.05).There was no significant difference in the number of people with no anxiety, mild anxiety, moderate anxiety and severe anxiety (P > 0.05). The postoperative SAS scores decreased in both the observation and control groups compared with the preoperative scores, but the preoperative SAS score decreased from 65.58 ± 8.20 to 53.03 ± 10.24 in the intervention group, while the preoperative SAS score decreased from 66.27 ± 9.73 to 60.87 ± 8.97 in the control group.The postoperative SAS score of the intervention group was significantly lower than that of the control group (*P* < 0.05). The number of people with no anxiety, mild anxiety, moderate anxiety, and severe anxiety were 21, 20, 13, 2 in the intervention groupand 4, 18, 18, 3 in the control group. The number of people with all degrees of anxiety in the intervention groupwas significantly reduced, and there was a significant difference compared with the control group (*P* < 0.05; Table 4).

### Doctor and patient satisfaction

The doctor and patient satisfaction scores of the intervention group were higher than those of the control group. The implementation of the checklist-based nursing care was effective in enhancing doctor and patient satisfaction with nursing care, and a significant difference was found between the two groups (*P* < 0.05; Table 5).

**Table 1:** The basic information between the two groups

Item	Intervention group (*n* = 56)	Control group (*n* = 53)	χ^2^ or t	*P*
Age($\bar x \pm s$, years)	65.05 ± 10.14	65.02 ± 10.73	0.017	0.986
Gender(cases)			0.371	0.542
Male	39	34		
Female	17	19		
Ethnicity (cases)			0.000	1.000
Han	55	52		
Other	1	1		
Marriage status (cases)			0.320	0.572
(Married)	55	50		
(Single)	1	3		
Profession(cases)			0.966	0.326
Retired	41	43		
On-the-job	15	10		
Medical Insurance Type(cases)			2.810	0.094
In the city	50	41		
Out of the city	6	12		
Days of hospitalization($\bar x \pm s$, days)	6.18 ± 2.39	6.85 ± 3.18	-1.260	0.210

**Table 2:** Comparison of preoperative care time between two groups of patients

Item	Intervention group (*n* = 56)	Control group (*n* = 53)	χ^2^ or t	P
Preoperative nursing care time (x ± s,mins)	37.61 ± 5.15	50.98 ± 5.61	-12.965	< 0.001
Preoperative nursing care time grouping			79.971	< 0.001
< 40 min	40	2		
40–50 min	16	20		
> 50 min	0	31		

**Table 3:** Comparison of the incidence of nursing-related adverse events in the two groups [n(%)]

Groups		Intervention group (*n* = 56) *n*(%)	Control group (*n* = 53) *n*(%)	χ2 value	*P* value
Preoperative	Intravenous Access Related	2(3.57)	7(13.21)	2.187	0.139
	Skin preparation in the operating area related	1(1.79)	6(11.32)	2.686	0.101
	Omission goods	1(1.79)	8 (15.09)	4.731	0.030
	Vomiting	35	2	0.001	0.978
Intraoperative	Defecation	16	20	0.567	0.451
Postoperative	Puncture site related	1(1.79)	3 (5.66)	0.320	0.572
	Difficulty in urination	0	2 (3.77)	0.567	0.451
	Skin Related	0	1 (1.89)	0.001	0.978
	Total occurrence	5(8.93)	30(56.60)	28.391	< 0.001

**Table 4:** Comparison of SAS scores in the two groups

Item	Intervention group (*n* = 56)	Control group (*n* = 53)	χ^2^ or t	*P*
Preoperative score (x ± s, scores)	65.58 ± 8.20	66.27 ± 9.73	-0.403	0.688
Preoperative score grouping			2.892	0.409
< 50	1	3		
50–59	12	11		
60–69	26	18		
> 69	17	21		
Postoperative score (x ± s, scores)	53.03 ± 10.24	60.87 ± 8.97	-4.236	< 0.001
Postoperative score grouping			20.471	< 0.001
< 50	21	4		
50–59	20	18		
60–69	13	18		
> 69	2	13		
t	11.624	4.782		
*P*	< 0.001	< 0.001		

**Table 5:** Comparison of doctor and patient satisfaction in the two groups (‾x ± s, scores)

Groups	Cases	Doctor satisfaction	Patient satisfaction
Intervention group	56	112.89 ± 12.11	93.64 ± 6.89
Control group	53	100.09 ± 8.34	84.32 ± 7.46
t		6.392	6.780
*P*		< 0.001	< 0.001

## Discussion

At present, there is no unified standard and consensus on the nursing care process before and after PCI procedures in China, and various medical units have discussed the nursing care methods for patients undergoing PCI procedures from different nursing perspectives [13, 26–30]. Although some foreign scholars have made Consensus Statement of Standards for Interventional Cardiovascular Nursing Practice [31], the ones for patients undergoing CTO intervention alone were not mentioned. Most of the research of these scholars is to improve the quality of life, psychological state and prognosis of patients, and few studies on the nursing work itself.Based on this, for nurses, effective clinical nursing work methods play a very important role in reducing nursing care time, improving work efficiency, reducing nursing-related adverse events, and improving doctor and patient satisfaction.Previous studies have shown that nursing intervention in the interventional treatment of chronic complete occlusion of coronary artery disease has a very significant clinical effect, not only can improve the success rate of surgery, but also can reduce complications, so as to make patients satisfied [32].

This study showed that the nurses in the intervention group spent significantly less time on preoperative nursing care than those in the control group.The nurses in the intervention groupused a preoperative nursing care checklist and completed the checklist item by item to ensure the continuity of nursing care. Despite interruptions in some special cases, the overall time spent on preoperative nursing care was significantly reduced; thus, nurses could be more consistent in providing care and could devote the time saved to other nursing tasks.

Adverse events related to nursing care in patients with CTO before and after surgery were mainly caused by poor instructions and work omissions by nurses. In this study, both groups of patients had a high incidence of preoperative adverse events, which could indirectly lead to corresponding intraoperative and postoperative adverse events. The rates of various adverse events in the intervention groupbefore, during, and after surgery were significantly lower than those in the control group, and none of the patients in theintervention group experienced intraoperative vomiting or defecation, nor did they have any skin problems related to defecation difficulties or pressure sores after surgery, indicating that the adoption of the checklist-based nursing care process resulted in more adequate preoperative preparation and ensured that the nurses did not miss patient care tasks. Moreover, the adoption of the checklist-based nursing care process helped reduce the incidence of nursing-related adverse events in CTO surgery.

Anxiety is common in patients undergoing PCI, and several factors can increase the risk of depression in patients [33], especially before PCI. Some studies have shown that the prevalence of anxiety and depression in patients is significantly increased one day before and after PCI, and this anxiety can affect the patient’s postoperative anxiety and should be addressed early [34, 35]. PCI in patients with CTO is more complicated than normal PCI procedures, which are more complex and takes longer time; therefore, preoperative anxiety is more severe in patients with CTO.

This study also showed that the patients in both groups had different anxiety levels before surgery and that their anxiety scores decreased after surgery. However, compared with the scores of the control group, the scores of the intervention group decreased more significantly. This finding indicates that after the application of the checklist-based nursing care process, the nurses provided holistic preoperative and postoperative nursing care to patients, the nursing care was more consistent, the preoperative preparation was adequate without omission, the education content was complete, and the postoperative health education and psychological nursing care for patients played a certain role in alleviating anxiety.Relevant reports proposed that the whole seamless link nursing intervention can shorten the time of regional collaborative treatment for patients of acute coronary syndrome, improve the prognosis, reduce the economic burden of patients, and improve the efficiency of acute coronary syndrome treatment [36]. The study found that CTO interventional therapy should not only pay attention to the success of surgery, but also pay attention to the safety of patients and reduce the pain of patients. The rich experience in intraoperative cooperation and interventional knowledge of catheter room nurses, their familiarity with the interventional therapy strategies and procedures of CTO, the manifestations of intraoperative complications and imaging characteristics, their understanding of the characteristics, functions and use of interventional instruments and their proficiency in emergency measures, can help us to have nursing foresight for possible intraoperative complications, so as to achieve early prevention and detection. Early treatment, in order to prevent and reduce the occurrence of complications, so that the surgical outcome is more successful [37].

Clinicians are the closest and most important partners of nurses, and the evaluation of nursing work by doctors has a strong reference, which is of great significance to improve nursing services, enhance nursing quality, and promote hospital development [38]. Patient centeredness is the purpose of nursing care, and patient satisfaction has always been an important indicator of nursing care. Nursing plays a key role in enhancing healthcare services and improving patient satisfaction by strengthening patient education and participation [39]. This study shows that the use of the proposed checklist can remind and guide nurses in providing comprehensive care to patients. After the implementation of checklist-based nursing care process, the preoperative nursing care guidance becomes more comprehensive, and preoperative preparation is more sufficient, which prevents adverse events that disrupt the surgical process. Postoperative nursing care is properly implemented, which not only enables patients to pass through the hospitalization period smoothly but also reduces the workload of doctors.

### Clinical implementation

The nursing checklist used in this study can be applied to the perioperative care of clinical CTO patients undergoing PCI. Nurses refer to the checklist to implement nursing measures for patients one by one, and mark the completed items before completion to avoid omissions and confusion. Its content is clear and concise, classified according to items and content, easy to operate, standardized and easy to implement, and standardized the nursing process of CTO patients, And it is concluded that using checklists to care for CTO patients can improve work efficiency and reduce the occurrence of adverse events.

### Research recommendation and future considerations

This study is the first time that Checklist-based Nursing has been applied to the care of patients undergoing CTO interventional operation. Therefore, more randomized controlled trials should be conducted using other feasible nursing methods to identify the best and most effective nursing methods for CTO interventional operation.

### Research strengths and limitations

In Chinese studies, studies on improving nurses’ work efficiency before and after PCI are rarely found in the literature, and there are even fewer nursing processes solely for CTO patients. Starting from the nurses themselves, this study is the first to apply the checklist to patient nursing for CTO interventional procedures to explore effective working methods that can reduce the nursing staff’s preoperative preparation time and reduce nursing errors. The main limitation of this study is that it was a single-center experiment, which reduces generalizability. The selected patients were patients with standard conditions of CTO interventional operation, and the excluded patients were not collected. It is stated that the clinical nurses using the checklist recorded the amount of time they spent on preoperative preparation of patients. There is therefore the potential for bias to be introduced, as the nurses were evaluating their own practice. Due to limitations in the research design, this study did not employ a fully random method, which may introduce certain selection bias.

CTO is a complex vascular disease, and studies have shown that nursing intervention in the interventional treatment of chronic completely occluded coronary artery disease can not only improve the success rate of surgery, but also reduce complications, making patients satisfied, which is consistent with the nursing intervention results used in this study. A systematic review [42] of safety checklists found that the safety of patients is not consistent and requires more high-quality research to confirm. But these articles only focus on exploring patient safety, and the studies involved are all historical controls, with bias and confounding. A randomized controlled trial found that the use of checklists was not meaningful in terms of mortality rates and improving nursing processes in critically ill patients. However, this study not only used checklists, but also conducted goal setting and physician prompted interventions, and did not mention the relationship between the use of checklists and improving nursing efficiency and reducing nursing defects. This study compared routine care with the use of checklists in nursing interventions for CTO patients. It was found that using checklists in nursing interventions for CTO patients can significantly shorten preoperative nursing time, reduce the occurrence of nursing defects, and reduce patient anxiety, improving patient and doctor satisfaction with nursing.

## Conclusion

The application of a checklist-based standard nursing care process in patients undergoing CTO interventions can significantly reduce the preoperative nursing care time, alleviate patient anxiety, improve patient and doctor satisfaction with the nursing care, and effectively reduce the occurrence of adverse events caused by nurses’ omissions or inadequate instructions. The proposed checklist-based standard nursing care process is worthy of clinical promotion and application.

## Data Availability

The datasets used and/or analysed during the current study are available from the corresponding author on reasonable request.

## References

[1] Lee SW, Lee PH, Ahn JM (2019). Randomized Trial evaluating percutaneous coronary intervention for the Treatment of Chronic Total Occlusion. Circulation.

[2] Koelbl CO, Nedeljkovic ZS, Jacobs AK (2018). Coronary chronic total occlusion (CTO): a review. Rev Cardiovasc Med.

[3] Allahwala UK, Ward MR, Brieger D, Weaver JC, Bhindi R (2019). Indications for percutaneous coronary intervention (PCI) in chronic total occlusion (CTO): have we reached a DECISION or do we continue to EXPLORE after EURO-CTO?. Heart Lung Circ.

[4] Roth C, Goliasch G, Aschauer S (2020). Impact of treatment strategies on long-term outcome of CTO patients. Eur J Intern Med.

[5] Park TK, Lee SH, Choi KH (2021). Late Survival Benefit of Percutaneous coronary intervention compared with medical therapy in patients with coronary chronic total occlusion: a 10-Year Follow-Up study. J Am Heart Assoc.

[6] Ladwiniec A, Allgar V, Thackray S, Alamgir F, Hoye A (2015). Medical therapy, percutaneous coronary intervention and prognosis in patients with chronic total occlusions. Heart Br Card Soc.

[7] Jeroudi OM, Alomar ME, Michael TT (2014). Prevalence and management of coronary chronic total occlusions in a tertiary Veterans affairs hospital. Catheter Cardiovasc Interv off J Soc Card Angiogr Interv.

[8] Fefer P, Knudtson ML, Cheema AN (2012). Current perspectives on coronary chronic total occlusions: the Canadian Multicenter Chronic Total Occlusions Registry. J Am Coll Cardiol.

[9] Christofferson RD, Lehmann KG, Martin GV, Every N, Caldwell JH, Kapadia SR (2005). Effect of chronic total Coronary Occlusion on treatment strategy. Am J Cardiol.

[10] Rigger J, Hanratty CG, Walsh SJ, Common, Uncommon CTO, Complications (2018). Interv Cardiol Lond Engl.

[11] Zhu T, Liu H, Han A, Gu H, Li X (2021). Orem’s self-care to treat acute coronary syndrome after PCI helps improve rehabilitation efficacy and quality of life. Am J Transl Res.

[12] Zhou Y, Wang X, Lan S, Zhang L, Niu G, Zhang G (2021). Application of evidence-based nursing in patients with acute Myocardial Infarction complicated with Heart Failure. Am J Transl Res.

[13] Lin C, Liu H, Liu X, Zhang Y, Wu F (2021). The application of whole-course nursing in patients undergoing emergency PCI and its impact on cardiac function. Am J Transl Res.

[14] Zhang Y, Chen G, Huang D, Luo S (2022). Clinical nursing pathway improves therapeutic efficacy and quality of life of Elderly patients with Acute Myocardial Infarction. Comput Math Methods Med.

[15] Hernández-Lloret CM, Fernández-Caminero G, González-González H, Espino-Díaz L, Álvarez-Castillo YJL (2021). Check-list and questionnaire datasets on diversity in Spain’s higher education. Data Brief.

[16] Lee KH, Chen TH, Shang G, Clulow S, Yang YJ, Lin SM (2019). A check list and population trends of invasive amphibians and reptiles in Taiwan. ZooKeys.

[17] Farage P, Puppin Zandonadi R, Cortez Ginani V, Gandolfi L, Yoshio Nakano E, Pratesi R. Gluten-Free Diet: from development to Assessment of a check-list designed for the Prevention of Gluten Cross-contamination in Food Services. Nutrients. 2018;10(9). 10.3390/nu10091274. 10.3390/nu10091274 PMC6165388 30201860

[18] Millat B (2012). The check list: a useful tool for the entire operation room team. J Visc Surg.

[19] Cavalcanti AB, Bozza FA, Machado FR (2016). Effect of a quality improvement intervention with Daily Round checklists, goal setting, and Clinician Prompting on Mortality of critically Ill patients: a Randomized Clinical Trial. JAMA.

[20] Indraccolo U, Graziani C, Di Iorio R, Corona G, Bonito M, Indraccolo SR (2015). External cephalic version for singleton breech presentation: proposal of a practical check-list for obstetricians. Eur Rev Med Pharmacol Sci.

[21] Takkavatakarn K, Puapatanakul P, Kanjanabuch T (2020). An early experience of check list to improve patient self-care and product defect report in continuous ambulatory peritoneal Dialysis (CLIP-SP) study. Int J Artif Organs.

[22] Purim KSM, Gonçalves CG, Binotto L (2019). Safety check list in outpatient Surgery teaching. Rev Col Bras Cir.

[23] World Medical Association (2013). Declaration of Helsinki: ethical principles for medical research involving human subjects. JAMA.

[26] Liu Q, Qi B, Zhang L, Zhang M, Xiao L. Effect of continuous nursing intervention on psychological state and medication compliance of patients with acute Myocardial Infarction after PCI. Panminerva Med Published Online September. 2020;18. 10.23736/S0031-0808.20.04077-X. 10.23736/S0031-0808.20.04077-X 32942835

[27] Feng J, Chen Y, Wang Y, Liu G. Application of establishing clinical nursing path in emergency PCI treatment of acute coronary syndrome. Panminerva Med Published Online June. 2020;18. 10.23736/S0031-0808.20.04012-4. 10.23736/S0031-0808.20.04012-4 32550628

[28] Guo W, Su Y, Chen L (2020). Effects of nursing methods for emergency pci and non-emergency PCI on the treatment of patients with acute Myocardial Infarction. JPMA J Pak Med Assoc.

[29] Li M, Liu H (2018). Implementation of a clinical nursing pathway for percutaneous coronary intervention: a prospective study. Geriatr Nurs N Y N.

[30] Qu B, Hou Q, Men X, Zhai X, Jiang T, Wang R (2021). Research and application of KABP nursing model in cardiac rehabilitation of patients with acute Myocardial Infarction after PCI. Am J Transl Res.

[31] White K, Macfarlane H, Hoffmann B (2018). Consensus Statement of standards for Interventional Cardiovascular nursing practice. Heart Lung Circ.

[32] Chen X (2016). Observation on the effect of nursing intervention in interventional treatment of chronic complete occlusion of coronary artery. Chin J Mod Drug Application.

[33] Doi-Kanno M, Fukahori H (2016). Predictors of Depression in patients diagnosed with Myocardial Infarction after undergoing percutaneous coronary intervention: a literature review. J Med Dent Sci.

[34] Gu G, Zhou Y, Zhang Y, Cui W (2016). Increased prevalence of anxiety and depression symptoms in patients with coronary artery Disease before and after percutaneous coronary intervention treatment. BMC Psychiatry.

[35] Trotter R, Gallagher R, Donoghue J (2011). Anxiety in patients undergoing percutaneous coronary interventions. Heart Lung J Crit Care.

[36] Zhao Y, Zhang W, Liang Y, Yan J, Cao S (2017). Application of seamless link nursing intervention in the treatment of acute coronary syndrome. Chin J Nurs.

[37] Xi A, Li S, Luan X, Xu N (2016). Risk prediction and nursing of postoperative Complications of chronic occlusive lesions. Nurs Pract Res.

[38] Ludman P, British Cardiovascular Intervention Society. BCIS audit returns. Adult interventional procedures January 2016 to December 2016. Available at Available at: www.bcis.org.uk/resources/audit-results/ (accessed 23 August 2018).

[39] Zhang T, Qi X (2021). Greater nursing role for enhanced post-percutaneous coronary intervention management. Int J Gen Med.

[40] McPherson S, Reese C, Wendler MC (2018). Methodology update: Delphi Studies. Nurs Res.

[41] Dunstan DA (2020). Scott N.Norms for Zung’s self-rating anxiety scale. BMC Psychiatry.

[42] Ko HC, Turner TJ, Finnigan MA (2011). Systematic review of safety checklists for use by medical care teams in acute hospital settings–limited evidence of effectiveness. BMC Health Serv Res.

